# Atmospheric aerosol-microplastics intake and deposition in the alveolar region by considering dynamic behavior of acinar airways

**DOI:** 10.1371/journal.pone.0327416

**Published:** 2025-08-20

**Authors:** Hamidreza Mortazavy Beni, Hamed Mortazavi, Puchanee Larpruenrudee, YuanTong Gu, Emilie Sauret, Mohammad S. Islam

**Affiliations:** 1 Department of Biomedical Engineering, Ars. C., Islamic Azad University, Arsanjan, Iran; 2 School of Mechanical and Mechatronic Engineering, University of Technology Sydney (UTS), Ultimo, New South Wales, Australia; 3 School of Mechanical, Medical and Process Engineering, Faculty of Engineering, Queensland University of Technology, Brisbane, Queensland, Australia; National Institute of Environmental Health Sciences, UNITED STATES OF AMERICA

## Abstract

**Background and objective:**

Atmospheric aerosols from different industrial and natural sources enter the airways during inhalation. The smaller respirable aerosols enter the alveolar sacs and, depending on the residence time and toxicity, create severe respiratory health hazards. The physiological movement of the alveolar sacs is an important feature of breathing dynamics. Therefore, the knowledge of the dynamic behavior of the alveolar airways during airflow and aerosol transport is essential for the accurate health risk assessment of respiratory aerosols.

**Methods:**

This study analyzed the physiological movements of the alveolar sac and its impact on airflow and particle deposition in the acinar region. In the present study, the dynamic acinar model uses a Computational Fluid-Particle Dynamics (CFPD). The boundary condition of moving walls is presented by introducing a novel strategic motion function of the alveoli (Eq. 5) compatible with the physiological function of the lung.

**Results:**

The results of the present study indicated that particle density is a determining factor in increasing the percentage of particle pollution deposition lower than 3 µm. The study also reports that the air amplitude velocity (~0.01 vs. 0.00085 m/s) is a crucial index in the particle pollution deposition in alveoli.

**Conclusions:**

To date, several studies analyzed the airflow in acinar sections. However, a comprehensive analysis of the physiological behavior of the alveolar sacs is missing in the literature. The specific findings of this study would improve the knowledge of airborne particle transmission in the alveolar zone.

## 1. Introduction

**Fig 1:**
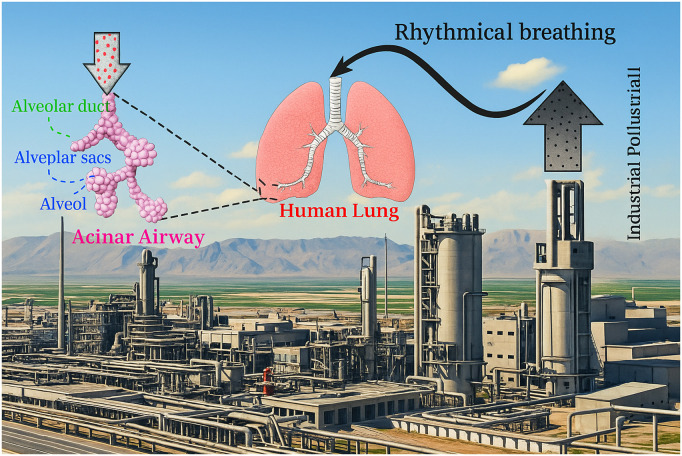
Graphical presentation of the intake of the industrial pollutant to acinar airways.

The amounts of respirable atmospheric aerosols are significantly increasing due to different natural and man-made activities. Inhalation of respirable airborne particles usually causes health problems. Airborne particulate matter not only cause lung diseases but also cardiovascular diseases. Increasing levels of air pollution due to urban development, by-products of industrial waste, and low air quality (Fig 1) at home and work are among the factors affecting human health. These factors can cause allergies, sinusitis, asthma, pulmonary emphysema, alveolitis, and lung cancer [1–5]. Many advances in particle deposition modeling have taken place with the development of medical equipment and image processing methods [6–10]. So high-quality images are exported by CT and MRI and used in modeling. In addition, measuring health risks is especially important in cases such as the prevalence of infectious diseases. For example, acute influenza outbreaks, such as bird and swine flu, and especially coronavirus, have recently spread to various variants. With the increase in air travel compared to the past, pathogens spread rapidly to distant areas and affect more people. In their research, Khan et al. [11] showed that airline passengers from March to April 2009 unknowingly transmitted the swine flu virus to various cities worldwide. Yan et al. [12], in their research using CFPD, found that emitted particles from the sneezing of people inside an aircraft are transmitted by the recirculation of the air inside the aircraft.

Studying airborne droplet inhalation to evaluate potential hazards to the respiratory system is often performed by predicting particle deposition using the CFPD. The beginnings of measured lung airway dimensions can be attributed to Weibel [13] and Horsfield et al. [14], who studied the field in 1963 and 1971, respectively, in separate models. These two models are the cornerstone of many studies on the deposition of particles in the respiratory tract through computational and experimental methods. Particle deposition also plays a crucial role in measuring health and predicting lung cancer progression, as shown by the previous work [15,16]. The use of CFPD in nasal cavity studies began later than in the respiratory tract with Elad et al. [17], who used a simple model in their research, followed by Keyhani et al. [18] from an actual model. In addition, many studies [19,20] have paid more attention to the deposition of ultrafine particles in the human respiratory tract. Oberdörster et al. [21] found that many inhaled particles are small and more penetrating in the respiratory system than larger particles of the same material. Also, Wang et al. [22] and Zamankhan et al. [23] modelled the deposition of ultrafine particles in the same trend. Kumar et al. [24] report that the movement of the wall creates a circulatory area in the respiratory bronchi and airway. With recent efforts [25], the correlation of particle deposition with the geometry of alveolar sacs was developed. Talaat and Xi [26] study showed that alveolar deposition was sensitive to particle diameter, alveolar orientation, and respiratory rate but not to respiratory depth.

Experimental and laboratory evaluation of gas exchange during respiration is always complicated. Inhalation and exhalation occur in the downstream areas of the lungs in acinar airways [27]. Anatomically, air enters the alveolar duct at the end of the airway pathway and eventually enters the alveolar sacs [28]. It is vital to investigate the effect of these particles by considering the possible dangers of particles entering the respiratory tract and transporting them to the alveolar areas. Although alveoli are usually depicted as spheres or small hemispheres attached to the end of the alveolar duct, they have also been modelled as hollow polyhedrons in previous studies [29]. Van Ertbruggen et al. [30] simulated the flow inside a curved alveoli model. Their findings showed that alveolar sacs have minimal effect on the mainstream in the central duct. Sznitman [31] modelled a three-dimensional geometry of the complete sections of a pulmonary acinar to study airflow movement. The results of this work also showed the existence of a rotation pattern in the direction of radial flow. Kumar et al. [32] used a multifaceted honeycomb geometry with flexible walls to measure fluid flow under rhythmical breathing. These results showed that the flow structure is strongly influenced by a progressive rotation zone with the ability to develop up to the third generation of the acinar region.

The present study presents a novel rhythmic function for the movement of the alveolar sac wall; it examines the alveolar behavior pattern and airflow associated with atmospheric particle injection in distal acinar airways under cyclic breathing conditions. In previous investigations, the flow and motion of particles within the flow field have been studied without elaborating on the motion function of the alveolar sacs [33–36]. The present study is the study that deals with the extent to which this particle deposition in the downstream acinar of the respiratory system. The respirable atmospheric aerosol deposition study in acinar is vital because the dangerous effects of toxic industrial aerosol exposure can be examined by identifying it. The effect on human health due to particle deposition in the alveolar sac is the focus of the present study.

## 2. Materials and methods

### 2.1. Geometry generation

**Fig 2:**
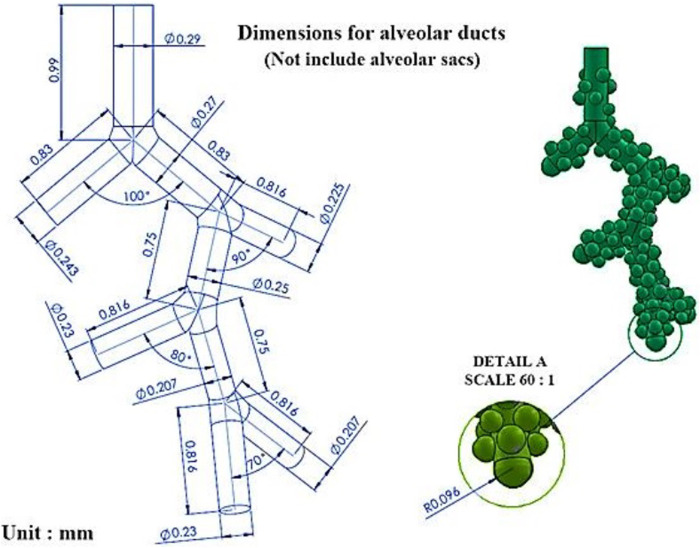
A five-generation asymmetric model which is compatible with actual acinar airway geometry.

Characteristics considered for the geometry of the model used in this research are chosen from the study of Haefeli‐Bleuer and Weibel [37]. Due to the limitations of the computer system in simulating complex geometries, some simplifications have been applied to it. These specifications are the actual dimensions of an acinar airway in an asymmetric five-generation model and are consistent with the morphometric data. Fig 2 provides the dimensions of alveolar ducts based on Haefeli-Bleuer and Weibel’s dimensions. No ethical clearance is required for the present model.

### 2.2. Governing equations for the flow-particle field

The governing equations of airflow are the equations of mass conservation and momentum, which are expressed as equations (1) and (2), respectively:

Continuity equation:


$$\vec{\nabla }.\vec{V}=0$$ (1)

Navier–Stokes equation:


$$\rho \frac{D\vec{V}}{Dt}=-\vec{\nabla }.P+\mu \nabla^{2}\vec{V}+{\vec{F}}_{g}$$ (2)

In these equations, V, ρ, μ, and $F_{g}$ are velocity, density, dynamic viscosity, and body force, respectively. This study used an incompressible laminar airflow with an unsteady state condition modelled by a sinusoidal velocity profile in the form of Eq. 3. Also, velocity amplitude 0.00085m/s has been selected based on previous studies [33,48]. According to Eq. 3, the breathing period is 4 seconds, so 2 seconds for inhalation and 2 seconds for exhalation. The movements of the alveoli are dependent on the airflow through the ducts and are driven by Eq. 3.


$$V_{\text{inlet\textbackslash{} velocity}}=0.00085\sin\frac{\pi }{2}t$$ (3)

In general, we can consider the function of changing the volume of sacs as Eq. 4. In this regard, $\forall_{ave}$ is the average volume of the sacs, and the value of 0.167 is based on a tidal volume of 0.5 liters, representative of normal resting respiration. Also, for the assumed functional residual capacity (FRC) of 3 litres, the volume excursions are 16.7% [33]. The frequency of Eq. 3 and Eq. 4 is considered the same ($\omega =\frac{\pi }{2}$). Physiologically speaking, the airflow creates airway wall expansion into the lung and through the airways and alveoli.


$$\forall =\forall_{\text{ave}}(1+0.167\sin\frac{\pi }{2}t)$$ (4)

Based on equation 4, the displacement function of the sac wall can be proposed as Eq. 5. So, $R_{ave}=0.063347mm$ is the average radius of the alveolar sacs. The fluid-structure interaction is defined by Eq. 5. As mentioned, the time period equality ($T=\frac{2\pi }{\frac{\pi }{2}}=4sec$) of Eq. 3 and Eq. 4 leads to that airflow forcing expanding alveolus. The dynamic deformation of the alveolar sac is implemented by Eq. (5) and is the dynamic equation applied to the alveolar sac boundary wall. It is average radius driven; physical deformation accounting for alveolar sac shape can be captured. This could affect the subsequent airflow and particle movement results.


$$\Delta \text{R}={\text{R}}_{\text{ave}}(\sqrt[{3}]{\left(1+0.167\sin\frac{\pi }{2}t\right)}-1)$$ (5)

The equation of motion of each particle is defined as Eq. 6. In this equation, $u_{i}^{p}$ is the instantaneous velocity of the particle, and its mass is calculated by multiplying the density $\rho^{P}$ by the particle volume $\forall_{P}$. On the right side of the equation are the forces acting on the particle, including the drag force ($F_{D}$), gravity term ($F_{g}$), Saffman’s lift force ($F_{L}$), and thermophoretic force ($F_{T}$). In most engineering problems, the critical force is the drag force applied to the particle by the surrounding fluid. Also, the lift force is significant only in turbulent flows. Brownian force is not considered because its effect is negligible for particles larger than 0.5 *μm* [38]. The Brownian motion could impact other deposition mechanisms, such as impaction.


$$\rho^{P}\forall_{P}\frac{du_{i}^{p}}{dt}=F_{D}+F_{g}+F_{L}+F_{T}$$ (6)

In the Discrete Phase Model (DPM), wall deposition can be monitored by particle erosion rate. It is defined as


$${\text{R}}_{\text{Erosion}}=\sum_{P=1}^{N_{particles}}\frac{{\dot{m}}_{p}F(d_{p})G(\alpha )V^{H(V)}}{A_{face}}$$ (7)

where $F(d_{p})$, $d_{p}$, G(α), α, H(V), V, and $A_{face}$ are a function of particle diameter, the particle diameter, a function of impact angle, the impact angle of the particle path with the wall face, a function of relative particle velocity, the relative particle velocity, and the area of the cell face at the wall, respectively. Particle erosion rate is an index that shows the amount of deposition mass on the surface. This makes it possible to identify geometry zones prone to the most deposition.

### 2.3. Numerical setup

In this research, particle diameters injected into the model are less than 5 µm. So, particles with these dimensions can reach downstream areas of the airways. The density of these particles is for two materials, water droplets (1000 *kg*/*m*^3^) and particle pollution (2400 *kg*/*m*^3^). The number of inlet particles is 54,270 based on the mesh grids, injected into the model at second 4 after starting breathing and for 2 seconds during the inhalation period. Therefore, the concentration profile is uniform at the inlet cross-section. After the airflow becomes stable, particles are injected into the model from the 4sec to the 6sec. Injections were only in 4–6 sec of the breathing cycle for inhalation. Some of the particles were exhaled following each exhalation of the breathing cycle. These inhalations (0–2 sec, 4–6 sec, 8–10 sec,...) and exhalations (2–4 sec, 6–8 sec, 10–12 sec,...) of breathing cycles were connected. The trap condition is applied as a boundary condition in the acinar walls so that whenever a particle hits the wall, it remains fixed at the same point of impact. Also, that point is considered the particle deposition position. Due to the macroscopic scale of the fluid particles, One-Way Fluid-Particle Interaction has been used [39]. The normal gravitational force of the earth is 9.8 *m*/*s*^3^ for the entire computational domain and its application to all particles [40]. Inhalation and exhalation periods continue until all particles in the mathematical model are deposited or escaped. The governing equations in this research are discretized by the finite volume method that can simulate complex geometric [41]. Ansys 2024 R1 software and laminar flow modules have been used in this research. The SIMPLE algorithm has been used in calculations in the Ansys Fluent software. According to the Courant number, the time step based on the physics of the present model in the simulation is equal to Δt = 0.01s. The result could be very sensitive to the time step for micron particles in the numerical study [45].

### 2.4. Grid independence test and validation of the results

**Fig 3:**
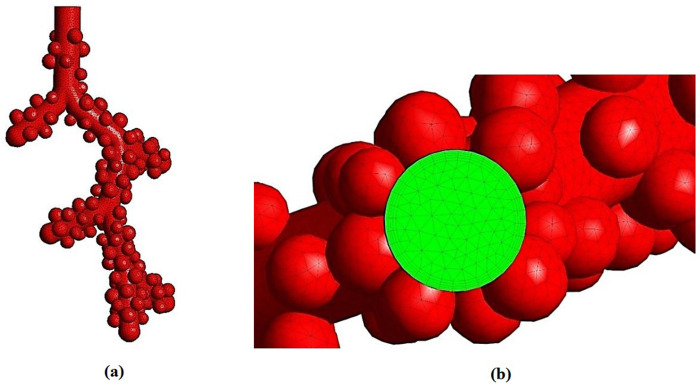
Generated grids on the human alveolar sacs from (a) side view and (b) top view.

Due to the importance of using a suitable computational mesh to solve numerical problems and the independence of the answers to the number and parameters used in the present study, this section investigated these feature limits. According to Fig 3, generated grids on the human alveolar sacs can be seen. Furthermore, the green zone is the inlet boundary condition in the acinar that is considered the cyclic flow rate.

**Fig 4:**
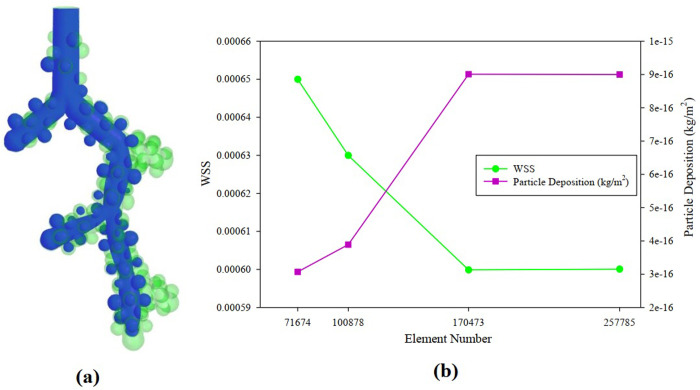
A grid size of 257,785 cells was obtained after the grid independence test. **(a)** Introduce the acinar middle plane (blue color). **(b)** Deposition fraction and WSS (wall shear stress) vs. Element Number curve on the acinar middle plane.

According to Fig 4, a grid size of 257,785 cells was obtained after the grid independence test. The deposition fraction results are less than a 1% deviation in the last two meshes on the middle blue plane (~ 258k vs. 170k).

**Fig 5:**
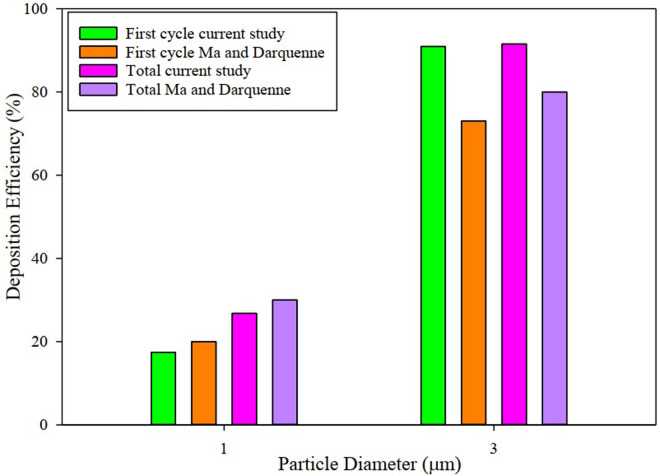
The verification model has successfully passed through the validation process [33].

By applying $\Delta R_{ave}$ to the walls of the sacs and using the dynamics mesh method along with particle injection, according to Fig 5, the verification model has successfully passed through the validation process, which is exactly the same as Ma and Darquenne [33] study in downward gravitational acceleration. Also, particle deposition history for 40 seconds and 1 *μm* particle injection are presented in Table 1 for better comprehension of the cyclic deposition pattern. As mentioned, inhalation of particles is just one time for inhalation periodic of 4–6 sec. Particle deposition and escape were obtained accumulatively after 40 sec. By 40 seconds, ten breathing cycles, including inhalation and exhalation, have been completed. By this time, the task of 99.8% of particles injected into the model has been determined. There is little difference between the present simulation results and the Ma and Darquenne [33] study because of the little difference between geometries.

**Table 1:** 30% deposition in the acinar airway model after approximately 40 seconds for 1 μm droplets.

time	trap	escape	trap+ escape	inject	Deposition
10s	7234	37698	44932	54270	0.133296481
12s	10466	37762	48228	54270	0.192850562
14s	12561	37762	50323	54270	0.231453842
16s	13641	37780	51421	54270	0.251354339
18s	14095	37780	51875	54270	0.259719919
20s	14591	37838	52429	54270	0.268859407
22s	15037	37838	52875	54270	0.277077575
24s	15399	37858	53257	54270	0.283747927
26s	15671	37858	53529	54270	0.288759904
28s	15866	37868	53734	54270	0.29235305
30s	16006	37868	53874	54270	0.294932744
32s	16092	37874	53966	54270	0.296517413
34s	16188	37874	54062	54270	0.298286346
36s	16243	37874	54117	54270	0.299299797
38s	16277	37874	54151	54270	0.299926294
40s	16320	37874	54194	54270	0.300718629

## 3. Results

This section presents the results obtained from modeling the acinar airway for the motion of particles in a transient velocity field. In the present study, by CFPD simulation using Ansys ® FLUENT 2024 R1, the boundary condition of moving walls was presented by introducing a novel strategic function (Eq. 5) of alveoli movement wall by implemented User Define Function (UDF) and injection of particles in the range of 0.5–5 µm. The analysis here applies to spherical particles. Also, evaporation and condensation of the particle in the human respiratory airway are ignored. The contours of this section are reported based on a droplet density of 1000 *kg*/*m*^3^. Also, the contours change for particle pollution with a density of 2400 *kg*/*m*^3^ with the same trend but in different quantities [51]. Finally, the deposition curve at the end of this section examines the deposition fraction difference between these two particles.

**Fig 6:**
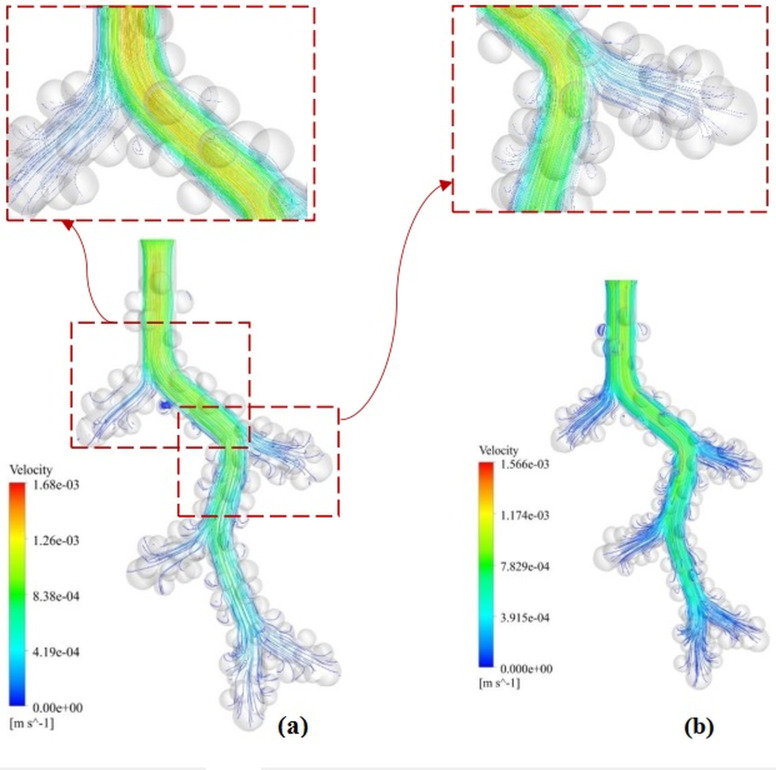
The air velocity streamlines in the acinar airway model. **(a)** 1 µm droplet inspiration. **(b)** 1 µm droplet expiration.

The air velocity streamline is shown in Fig 6. At the time of inhalation, the maximum velocity is in the centerline of the alveolar duct. Also, the velocity is reduced to zero in the areas close to the wall and deep in the alveoli. These characteristics are due to the fully developed airflow, consistent with the normal airflow state in the human respiratory tract. In the fully developed fluid flow, the maximum velocity is in the centre of the alveolar duct with an average amount of 0.00084 m/s, reaching zero in the walls. After that, the streamwise direction profile does not change [42,43]. However, the velocity profile varies near the alveoli due to the incoming airflow. On the other hand, the airflow is accompanied by a recirculation zone in some alveolus on exhalation.

**Fig 7:**
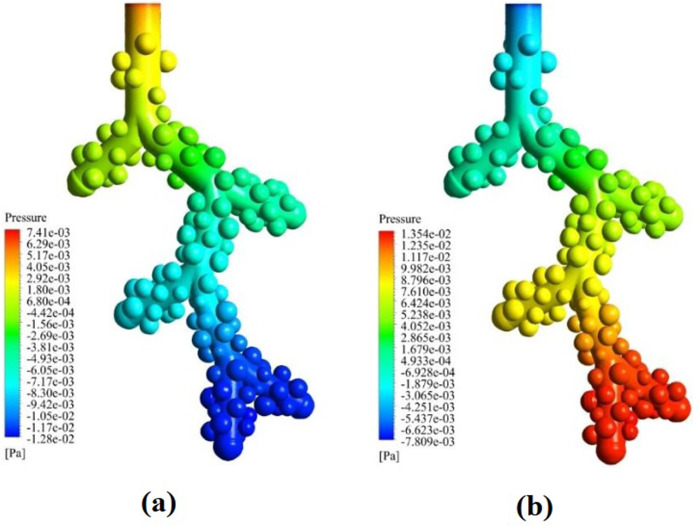
Pressure variation in the acinar airway model. **(a)** 1 µm droplet inspiration. **(b)** 1 µm droplet expiration.

The pressure fills contour is shown in Fig 7, which is compatible with the changes in the velocity contour. The airflow into the lung is the result of the difference in pressure at the mouth (atmospheric) and pleural cavity (negative). It should be negative average all throughout the lung during inhalation and above atmospheric during exhalation. However, the results here show this is due to correct boundary conditions. During inspiration, the highest relative pressure is at the alveolar duct inlet, and the lowest relative pressure occurs at the distal acinar airway, with an average amount of −0.0128 Pa. Also, during expiration, the lowest relative pressure is at the alveolar duct inlet, and the highest relative pressure occurs at the distal acinar airway, with an average amount of 0.0135 Pa. The velocity profile in fully developed laminar flow remains unchanged in the direction of fluid movement. Also, the fluid particles are moved in a state of constant axial velocity. In addition, there will be no movement in the radial direction, and the velocity component will be zero in all positions in the direction normal to flow.

**Fig 8:**
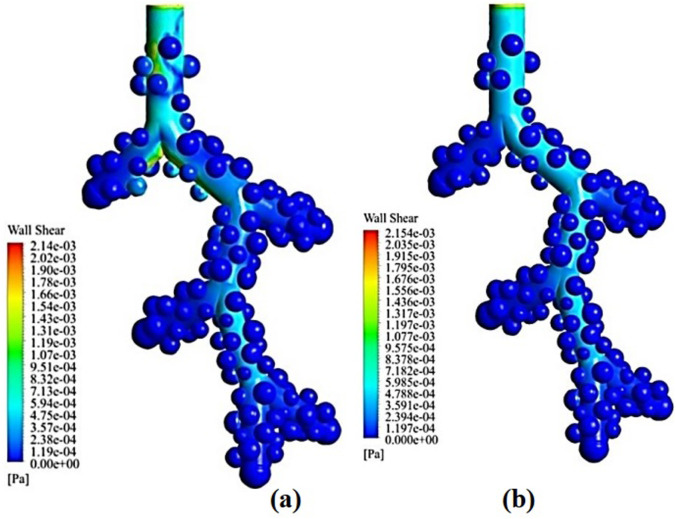
WSS variation in the acinar airway. **(a)** 1 µm droplet inspiration. **(b)** 1 µm droplet expiration.

Fig 8 shows the wall shear stress (WSS) in the model geometry. The results show that the shear stress inside the alveoli is very low but reaches its maximum in the central axis of the alveolar duct with an average amount of 0.00718 Pa and the areas where the alveoli connect to the central alveolar duct. Excessive WSS at the alveolar lining can damage the alveolar epithelial layer [44]. The epithelial lining is a portion of the alveolar membrane that permits the exchange of gases.

**Fig 9:**
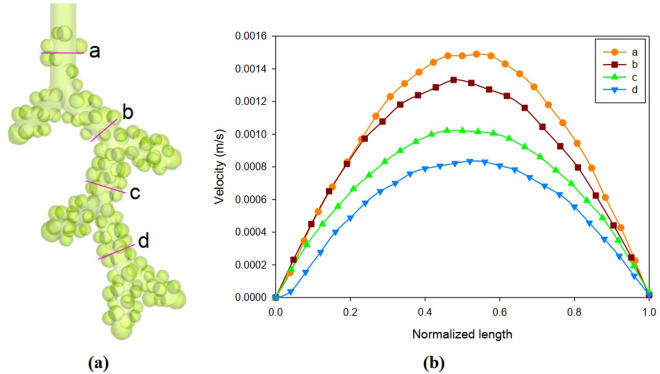
The air velocity profile for inhalation based on lines in each central alveolar duct generation at peak inhalation. **(a)** Cross-section Lines. **(b)** Velocity profile for selected locations.

The air velocity profiles at selected locations of the alveolar duct are presented in Fig 9 at peak inhalation. Also, Fig 9b presents some velocity profiles for inhalation based on Fig 9a cross-section lines in each central alveolar duct generation. The velocity profile in the alveolar duct is compatible with a fully developed laminar flow. Also, lower velocity amplitude exists closer to the distal acinar airway. The velocity profile for exhalation is similar to the inhalation quantities with a reverse direction. The average pressure drop in the four cross-sections of Fig 9a, which is related to the model inlet airflow, is shown in Table 2. The quantitative value of the average pressure drop in the four planes is almost the same. Also, most pressure drop is related to the distal acinar airway.

**Table 2:** The mean pressure drop in the four cross-sections of Fig 9a relative to the model inlet airflow.

Pressure Drop (Pa)	Plane a	Plane b	Plane c	Plane d
Inspiration	−0.003247	−0.007366	−0.012326	−0.015976
Expiration	0.003247	0.007434	0.012386	0.016252

**Fig 10:**
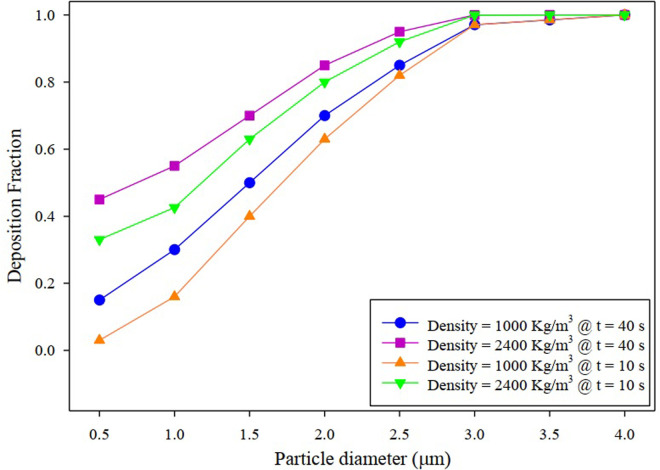
Deposition fraction variation versus particle diameter in the acinar airway.

The deposition diagram versus particle diameter is shown in Fig 10. Deposition fraction is the ratio of the number of particles depositing to the number of particles entering the alveolar model. As the droplet diameter increases, the particle deposition rate increases so that at a droplet diameter of 4 µm, the deposition rate is 100% due to the larger particle size. Also, for droplets at 0.5 µm, the amount of deposition is minimal. With increasing particle density up to 2400 *kg*/*m*^3^ for particle pollution, the amount of deposition increases due to the heavier particles so that at 3 µm, the deposition percentage is almost 100%. The trend of variation in the deposition curve at 10 and 40 seconds of the breathing cycle is almost the same. Also, as reported in Table 1, all the particles in the model are either trapped or escaped in about 40 seconds.

As the distal acinar airways are the last stage of the inhalation pathway, the upstream filtration disturbances will have a profound effect on the amount and physical profiles of particles that can enter this region. Particle injection in distal acinar airways can represent the physical reality of the respiratory and particle transport process, and many of the important physical events downstream exist. Therefore the present study has solid ground to justify its validity. The study of the deposition of toxic particles in the distal acinar airways has a special place. On the other hand, studying drug delivery in this area by improving the modeling method and increasing the accuracy of laboratory measurements can lead to significant effectiveness in this field related to human health.

## 4. Discussion

**Fig 11:**
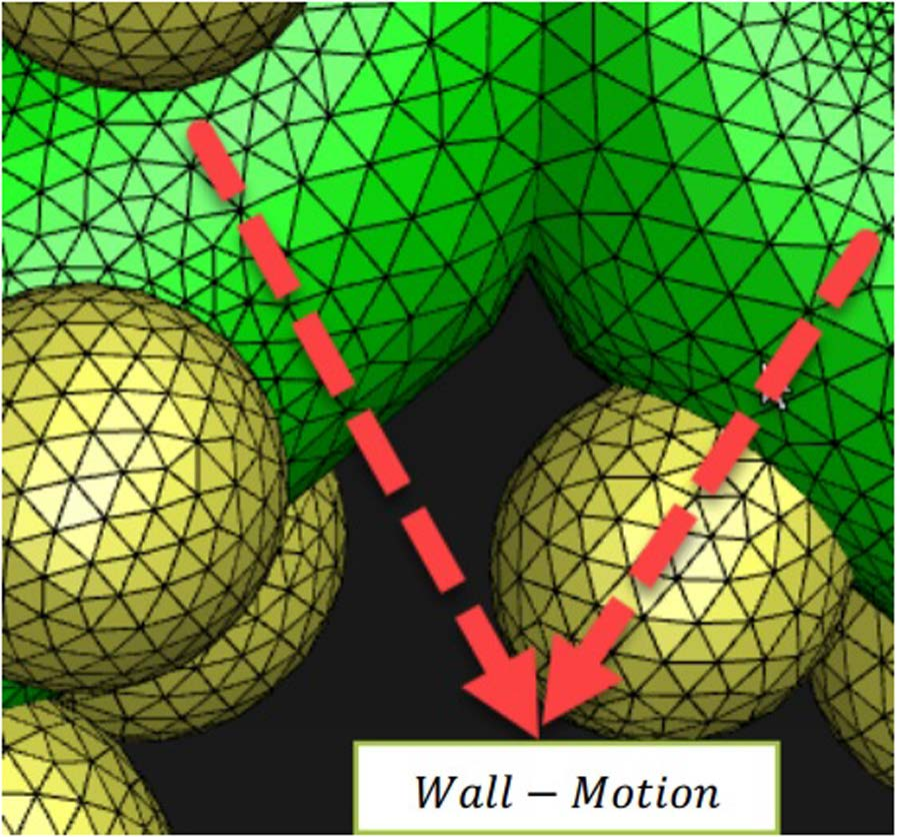
The alveolar sacs model that has been used with the equation of motion of the wall grid displacement as Eq. 5.

In the present study, the human acinar airway was modelled to identify particles and the effect of their downstream deposition on human health, the five-generation asymmetric acinar airway of the respiratory tract (Fig 2). In this regard, the effect of particle diameter and density on the deposition has been investigated. This can be useful in separating the effect of particles in pollutants with different diameters on the human lung. In the theory of dynamic deforming mesh, one equation must be applied to move the sac wall. In the present study, the dynamics mesh using the alveolar sacs motion function (Eq. 5) in a geometry consistent with reality tries to understand the flow behaviour patterns in the pulmonary acinar. According to the greened walls in Fig 11, interference and error are possible if the wall motion function is not defined correctly due to excessive enlargement of the sacs. This issue should be given special attention in introducing the wall motion function.

**Fig 12:**
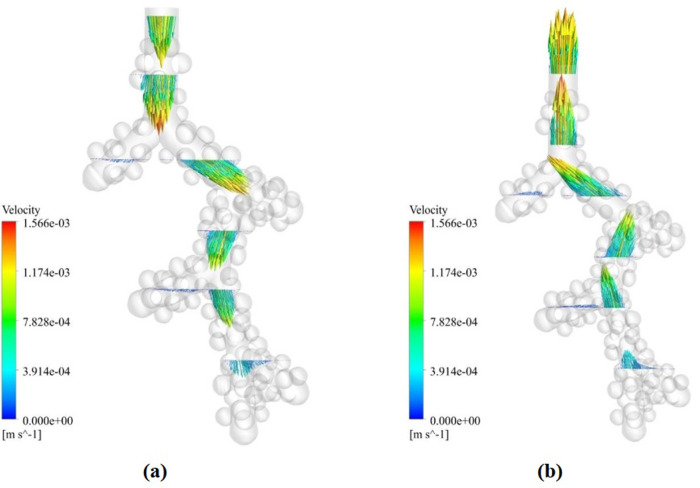
Velocity vector variation in the acinar airway in the cross-sectional plane. **(a)** 1 µm droplet inspiration. **(b)** 1 µm droplet expiration.

As observed (Fig 9), the velocity profile in the longitudinal distance between the alveoli is fully developed. Also, the flow representation that these lines shows the laminar flow profile based on reality. Therefore, its velocity and distribution are by the developed flow. Also, the velocity field at distances close to the wall is minimal and about zero, which is to the actual flow behavior and shows the correctness of the simulation in the dynamic mesh method. The parabolic shape of the velocity in the ducts is due to the uncoupling of the airflow through the airways and wall expansion. As a result, a sinusoidal inlet velocity (Eq. 3) leads to a parabolic (or better described by a sinusoidal) profile in the airways. Therefore, the particle diffusion model is completely influenced by the development of the fluid field, and the particles are dispersed according to the same pattern. Also, the particle diffusion model is affected by drag and gravity, and some particles are deposited by changing the direction of motion (Fig 12).

**Fig 13:**
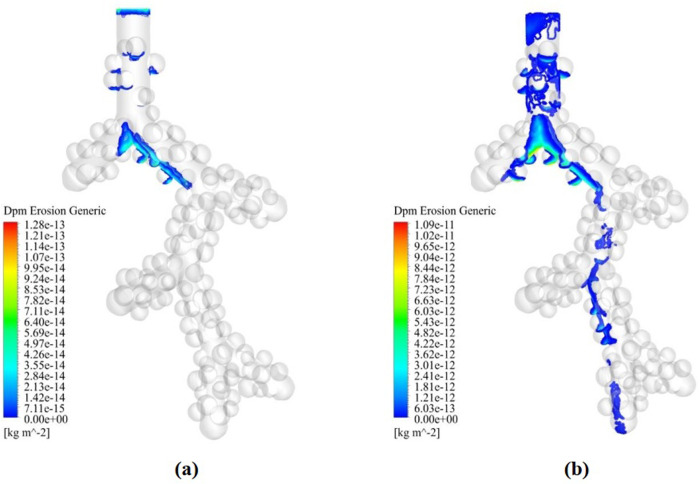
Particle deposition contour in the acinar airway. **(a)** 1 µm droplet inspiration. **(b)** 3 µm droplet inspiration.

Fig 10 showed that the particle diameter of the larger particles led to the steeper slope of the deposition diagram, which indicates the dependence of the deposition fraction on the diameter of the particles. The droplet deposition contour with particle diameters of 1 and 3 μm is shown in Fig 13. As can be seen, most of the concentration of droplet deposition occurs due to the collision with the first bifurcation, and overall deposition in the alveolus is negligible. In Fig 13b, almost all the droplets are located in the centerline of the alveolar duct. It can be attributed to droplets following the fluid direction in Reynolds <1. Because at Re < 1, the viscous forces of the fluid overcome the forces of inertia so that the droplets follow airflow streamlines closely. The present study used a tidal volume of 0.5 liters, representing normal resting inhalation. Therefore, according to Fig 13, particle deposition in the alveoli is the lowest in normal resting inhalation. At this distal acinar, inertia plays an important role.

**Fig 14:**
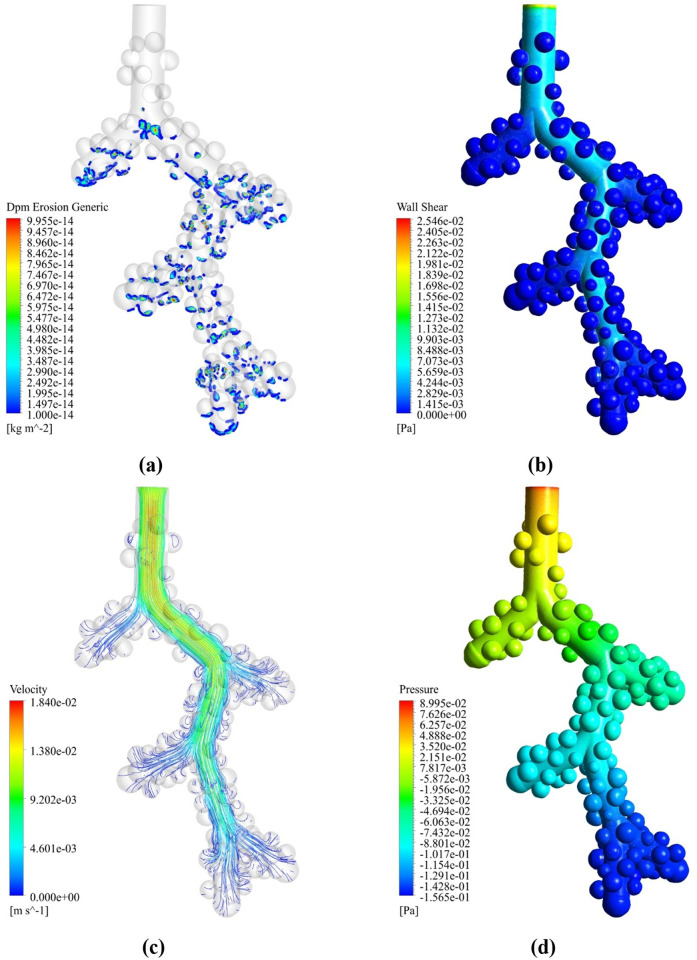
Deposition and WSS contours in the acinar airway with the 0.01 m/s velocity amplitude at 1 µm droplet inspiration. **(a)** Particle deposition contour. **(b)** WSS contour. **(c)** Velocity streamline. **(d)** Pressure counter.

In the present study, the velocity amplitude of the input airflow to the model is considered 0.00085 m/s based on the previous studies [33,44]. Increasing the velocity amplitude to 0.01 m/s by fast deep inhalations not only increases the percentage of droplet deposition but also increases the penetration of droplets in the distal acinar airways of the respiratory system (Fig 14a) [51]. The airflow rate is significantly reduced at the distal tracheobronchial branch of μm size. A rigorous calculation is needed to justify the airflow amplitude chosen for the study. With increasing air velocity amplitude, inertial forces overcome the viscous forces of the fluid so that droplets will detach from the airflow. The deposition in this status is 48% at 10 seconds, which is 3 times the deposition in the case where the inlet velocity amplitude was 0.00085 m/s. At this distal acinar, uniform diffusion in all directions is more reasonable. By increasing the velocity amplitude up to 0.01 m/s, the value of WSS (Fig 14b) also increases compared with a velocity amplitude of 0.00085 m/s. As mentioned, immoderate WSS at the alveolar lining can damage the alveolar epithelial layer [44]. The fluid velocity streamlines moved well in all alveolar sacs and covered those (Fig 14c). The highest pressure with a maximum positive value occurs at the inlet before the first alveolar bifurcation, and the lowest pressure with a minimum negative value occurs at the distal acinar airways (Fig 14d).

Investigation and evaluation of risk factors for the human respiratory system are of great importance in the health and survival of each individual. In recent years, in several types of research to study the transfer of aerosols and their deposition in the human respiratory tract, the computational fluid dynamics (CFD) has been used [12,18,20,30,32,45–48]. Some recent studies have also focused on the upper respiratory tract by the fluid-structure interaction, which has less wall deformation effect [49–52]. Still, only a few of these studies have focused on the alveolar region of the lung due to difficulties in applying the dynamic mesh method [30,47,52,53]. In these studies, a two-dimensional model with axial symmetry with rigid walls was used in a range of tubes, with one alveolus and a tube covered with alveoli throughout. Subsequently, recent studies have used dynamic walls [33,54]. Despite the low Reynolds number in the alveolar region (less than 1), these studies showed that flow in the alveolar region is very complex due to the time-dependent and unique geometry of the acinar airway. The transfer and deposition of aerosols are influenced by geometric features, especially at the alveolar diaphragm level. There are many heterogeneities in particle deposition patterns in the acinar airway. Including the alveolar septum increases the surface area available for particle deposition and thus modifies the concentration of particle deposition per unit area. On the other hand, considering the boundaries of the movable wall increases the convective exchange between the lumen and the surrounding alveolar reservoirs. For this reason, moving walls have been used in the simulation to get closer to the in vivo situation. Overall, the results of this study confirm that the size and concentration of particles are the dominant mechanisms in the deposition of particles in the distal acinar airways. Above all, the increase in air velocity in the downstream areas causes the deposition of particles in the alveolar sacs. So if the velocity amplitude (see Eq. 3) increased to 0.01, the penetration and deposition of particles in the alveolar raised. Also, the present study shows that particle deposition rate decreases sharply, especially with decreasing the diameter of particles. Having accurate information about the geometry of the lung [55] and then using numerical methods to simulate the function of the respiratory system [56] can provide a more precise understanding of medical therapy predictions [57,58] in the acinar airway.

## 5. Conclusions

The present study presents a rhythmic function for the movement of the alveolar sac walls. The impact of the dynamic movement of the alveolar sacs on airflow dynamics and particle deposition is investigated. An advanced modeling dynamic model for the acinar airways is presented. The key findings of the present study are listed below:

- The deposition rate increases with the diameter of the aerosol. The numerical results also report that most particles tended to deposit in the centerline of the alveolar duct;
- The junction of the alveoli with the central alveolar duct has the highest shear stress, which is essential in studying the mechanobiology of lung diseases;
- The computation study reports that density influences acinar deposition, and denser particles in 3 µm reach the highest rate of deposition in the acinar region;
- Particle deposition contours for the discrete phase are investigated. The numerical results show that increasing the velocity amplitude to 0.01 m/s by fast deep inhalations increases the deposition in the distal acinar airways. With increasing air velocity amplitude, inertial forces overcome the viscous forces of the fluid so that particles will detach from the airflow;
- The droplet deposition hot spot zone is investigated, potentially will benefiting the targeted drug delivery for future study.

The present study analyzed the dynamic behavior of the acinar airways. The comprehensive analysis of the flow field and particle transport results would improve the knowledge of the aerosol deposition pattern in the alveolar region. The findings of this study could potentially improve the knowledge of the field, which would benefit the design of the targeted drug delivery tools. The future study would consider the patient-specific model for the acinar airways and investigate the physiological behavior under disease conditions.
